# Challenges and opportunities for improving the landscape for Lewy body dementia clinical trials

**DOI:** 10.1186/s13195-020-00703-5

**Published:** 2020-10-29

**Authors:** Jennifer G. Goldman, Leah K. Forsberg, Bradley F. Boeve, Melissa J. Armstrong, David J. Irwin, Tanis J. Ferman, Doug Galasko, James E. Galvin, Daniel Kaufer, James Leverenz, Carol F. Lippa, Karen Marder, Victor Abler, Kevin Biglan, Michael Irizarry, Bill Keller, Leanne Munsie, Masaki Nakagawa, Angela Taylor, Todd Graham

**Affiliations:** 1 Parkinson’s Disease and Movement Disorders Program, Shirley Ryan AbilityLab and Departments of Physical Medicine and Rehabilitation and Neurology, Northwestern University Feinberg School of Medicine, 355 E. Erie Street, Chicago, IL 60611 USA; 2 Department of Neurology, Mayo Clinic, Rochester, MN USA; 3 Department of Neurology, University of Florida College of Medicine, Gainesville, FL USA; 4 Department of Neurology, Hospital of the University of Pennsylvania, Philadelphia, PA USA; 5 Department of Psychiatry and Psychology, Mayo Clinic, Jacksonville, FL USA; 6 Department of Neurosciences, UC San Diego, San Diego, CA USA; 7 Department of Neurology, University of Miami Miller School of Medicine, Miami, FL USA; 8 Department of Neurology, University of North Carolina, Chapel Hill, NC USA; 9 Lou Ruvo Center for Brain Health, Neurological Institute, Cleveland Clinic, Cleveland, OH USA; 10 Department of Neurology, Thomas Jefferson University, Philadelphia, PA USA; 11 Department of Neurology, Taub Institute, Sergievsky Center, Columbia University Irving Medical Center, New York, NY USA; 12 Acadia Pharmaceuticals, San Diego, CA USA; 13 Neuroscience Research, Eli Lilly and Company, Indianapolis, IN USA; 14 Neurology Business Group, Eisai, Inc., Woodcliff Lake, NJ USA; 15 Neurology Business Group, Eisai Co., Ltd., Tokyo, Japan; 16 Lewy Body Dementia Association, S.W., Lilburn, GA USA

**Keywords:** Biomarker, Clinical trial readiness, Dementia, Lewy bodies, Neuropsychology, Outcome measure, Parkinson’s disease, Parkinsonism, Primary endpoint, Randomized controlled trial

## Abstract

Lewy body dementia (LBD), including dementia with Lewy bodies and Parkinson’s disease dementia, affects over a million people in the USA and has a substantial impact on patients, caregivers, and society. Symptomatic treatments for LBD, which can include cognitive, neuropsychiatric, autonomic, sleep, and motor features, are limited with only two drugs (cholinesterase inhibitors) currently approved by regulatory agencies for dementia in LBD. Clinical trials represent a top research priority, but there are many challenges in the development and implementation of trials in LBD. To address these issues and advance the field of clinical trials in the LBDs, the Lewy Body Dementia Association formed an Industry Advisory Council (LBDA IAC), in addition to its Research Center of Excellence program. The LBDA IAC comprises a diverse and collaborative group of experts from academic medical centers, pharmaceutical industries, and the patient advocacy foundation. The inaugural LBDA IAC meeting, held in June 2019, aimed to bring together this group, along with representatives from regulatory agencies, to address the topic of optimizing the landscape of LBD clinical trials. This review highlights the formation of the LBDA IAC, current state of LBD clinical trials, and challenges and opportunities in the field regarding trial design, study populations, diagnostic criteria, and biomarker utilization. Current gaps include a lack of standardized clinical assessment tools and evidence-based management strategies for LBD as well as difficulty and controversy in diagnosing LBD. Challenges in LBD clinical trials include the heterogeneity of LBD pathology and symptomatology, limited understanding of the trajectory of LBD cognitive and core features, absence of LBD-specific outcome measures, and lack of established standardized biologic, imaging, or genetic biomarkers that may inform study design. Demands of study participation (e.g., travel, duration, and frequency of study visits) may also pose challenges and impact trial enrollment, retention, and outcomes. There are opportunities to improve the landscape of LBD clinical trials by harmonizing clinical assessments and biomarkers across cohorts and research studies, developing and validating outcome measures in LBD, engaging the patient community to assess research needs and priorities, and incorporating biomarker and genotype profiling in study design.

## Introduction

Lewy body dementia (LBD), comprised of dementia with Lewy bodies (DLB) and Parkinson’s disease dementia (PDD), affects approximately 1.4 million in the USA and carries substantial public health impact [1, 2]. Currently available symptomatic treatments for LBD vary in their effectiveness and symptom target (i.e., cognitive impairment, parkinsonism, psychosis, among others). Disease-modifying and curative treatments are lacking. Clinical trials for LBD are prioritized in the Alzheimer’s Disease (AD)-Related Dementia (ADRD) Summit recommendations [3], but randomized clinical trials in LBD are few, particularly compared to AD and Parkinson’s disease (PD) without dementia [4, 5]. In order to advance the field of LBD clinical trials, the Lewy Body Dementia Association (LBDA) developed a Research Centers of Excellence (RCOE) program in 2017, bringing together expert clinicians and researchers across the USA with a goal of becoming a clinical trials-ready network [6]. In 2019, the LBDA launched the Industry Advisory Council (IAC) to provide a collaborative forum for discussion among LBD experts, pharmaceutical industries, governmental agencies, and the nonprofit LBDA to address challenges and opportunities for LBD clinical trials. The inaugural LBDA IAC meeting, held in June 2019, focused on key gaps and challenges in clinical trial design and implementation in LBD. In this review, we discuss the formation of the IAC, current state of LBD clinical trials, and challenges and opportunities for optimizing future clinical trials in LBD.

## The need for partnerships and formation of the LBDA IAC

Over the years, partnerships have developed among academic medical experts, patient advocacy groups, pharmaceutical industries, and government bodies for neurological and other diseases to address clinical and research challenges. These collaborations have increased awareness and diagnosis of the condition, improved access to expert care, fostered interest for drug development, and promoted clinical trial site readiness, all critically important for increasing the likelihood of recruitment and completion of clinical trials [7, 8]. These partnerships may help reduce barriers in clinical research such as shortening the long latency between drug development and Food and Drug Administration (FDA) approval, reducing the frequency of failed drug trials, and potentially lessening the economic investment for drug development [9]. As such, the LBDA formed an Industry Advisory Council including invited members from the RCOE network and Scientific Advisory Council with clinical and research expertise in movement disorders neurology, cognitive neurology, and neuropsychology; pharmaceutical industries known to be currently sponsoring or considering phase 2 or 3 clinical trials for LBD; governmental agencies; and the LBDA foundation.

## Diagnostic criteria and standards of care for LBD

LBD remains underdiagnosed or misdiagnosed, which can delay appropriate clinical management and presents challenges for LBD clinical trials. Difficulty in symptom recognition, fluctuations in cognitive and functional presentations, and differences in specialty access and evaluations across healthcare systems contribute to these challenges [10–12]. The DLB diagnostic criteria, revised in 2017, define essential cognitive and core clinical features and provide a framework to incorporate indicative and supportive biomarkers of diagnosis [13]. The clinical diagnostic criteria for PDD, published in 2007, include features that distinguish dementia in the context of established PD from AD or other dementias [14]. Both DLB and PDD diagnoses are listed in the Diagnostic and Statistical Manual of Mental Disorders (DSM)-5 as major neurocognitive disorders [15]. Challenges and controversy, however, remain in dividing LBD into DLB and PDD given overlapping clinical symptoms, biomarker findings, and underlying pathology, with the “1-year” rule being a primary distinguishing factor. In addition, criteria for mild cognitive impairment (MCI) in PD and in DLB have been proposed [16, 17].

Additional tools have been developed to assist with diagnosing and characterizing DLB since past diagnostic criteria historically have had variable sensitivity in clinical practice (12–88%) [18]. These tools include the Lewy Body Composite Risk Score (LBCRS) [18], LBDA’s diagnostic symptom checklist [19], National Alzheimer’s Coordinating Center (NACC) DLB module [20], and DIAMOND-Lewy Toolkit [10, 21]. The LBCRS, a relatively short questionnaire and validated in a dementia population, discriminates between DLB and AD and between MCI in these two groups. The LBDA checklist provides a symptom list that people diagnosed with LBD or their caregivers can bring to a physician and a summary of DLB criteria. The NACC DLB module, developed in 2016 to accompany the Uniform Data Set used in NIA AD Centers, aims to better characterize LBD with a standardized clinical and cognitive battery. The DIAMOND-Lewy Toolkit was developed in the UK to assist with diagnosing DLB in clinical practice and uses brief assessments deemed feasible by dementia and PD clinicians. Harmonizing clinical assessment tools and application of diagnostic criteria across DLB and PDD cohorts in observational studies and across international clinical and research efforts remains a priority and is critical to LBD clinical trials.

At present, there is no consensus for a standard of care for LBD, and evidence-based strategies are limited. The UK DIAMOND-Lewy Management Toolkit provides clinicians with a management overview, symptom management summaries, and reference guide, and several recent reviews highlight management strategies [22–25]. Regularly updated evidence-based reviews are available for PD motor and non-motor symptoms, but are lacking in LBD, especially for DLB [26]. Medications for dementia have been studied in several double-blind, placebo-controlled studies for LBD, with donepezil approved for DLB in Japan and rivastigmine approved for PDD in the USA and European Union. Clinical care of LBD is complex due to a wide range of symptoms (cognitive impairment/dementia, parkinsonism, mood disorders, psychosis, apathy, autonomic dysfunction, sleep disturbances, and fluctuations), and treatment of one or more of these clinical features may exacerbate existing symptoms or produce new ones. The heterogeneity of LBD symptoms and presentations not only make a uniform standard of care difficult, but also underlie some clinical trial design challenges regarding study focus, subject criteria, and outcome measures.

## Current state of clinical trials in LBD

Despite the emphasis in NIH ADRD recommendations and increased numbers of clinical trials for LBD in recent years, randomized controlled trials in LBD lag behind those conducted in other neurodegenerative diseases (e.g., AD, non-demented PD), do not yet address the full range of LBD symptoms, and primarily focus on symptomatic rather than disease-modifying therapies. Several reviews discuss recent clinical trials in LBD [4, 5, 22, 24, 27]. In one review of DLB trials listed in ClinicalTrials.gov from 2002 to 2019, there were 87 registered trials, and of those, 30 trials studied pharmacological agents or devices [5]. There were 9 pharmacological agents and one device studied in 22 trials. Most trials were phase 2 or post-marketing or exploratory studies with a smaller number of phase 2/3 or pilot device studies. All focused on cognition, psychosis, or sleep symptoms, and one trial focused on motor symptoms. Fewer than 10 trials in LBD have been completed between 2016 and 2020, and recent/ongoing LBD trials remain largely focused on cognitive outcomes but may utilize novel or repurposed agents (Table 1). To date, no trials have studied fluctuations, dysautonomia, agitation, or apathy in LBD.

**Table 1:** Selected recent/ongoing and completed LBD clinical trials 2016–2020

ClinicalTrials.gov identifier	Population	Drug/intervention	Mechanism	Trial design	Primary outcome	Results for primary outcome
NCT03305809	DLB or PDD	Mevidalen	D1 positive allosteric modulator (D1PAM)	Phase 2, DB-PC	CDR computerized cognition battery continuity of attention composite score	Ongoing
NCT03413384	PDD	Ceftriaxone	Glutamatergic activity, excitotoxicity reduction	Phase 2, DB-PC	ADAS-Cog	Ongoing
NCT02914366	PDD	Ambroxol	Raise beta-Gcase, lower α-synuclein	Phase 2, DB-PC	ADAS-Cog, ADCS-CGIC	Ongoing
NCT03774459	PDD	Anavex2-73	Cellular homeostasis restoration via sigma-1 and muscarinic receptors	Phase 2, DB-PC	CDR computerized cognition battery continuity of attention composite score, safety	Ongoing
NCT03713957	PDD or PD-MCI	GRF6021	Plasma-derived product	Phase 2, DB-PC	Safety	Ongoing
NCT03467152	DLB	E2027	Selective phosphodiesterase inhibitor type 9	Phase 2, DB-PC	MoCA, CIBIC+	Ongoing
NCT04002674	DLB	Nilotinib	Tyrosine kinase inhibitor	Phase 2, DB-PC	Safety, tolerability	Ongoing
NCT02669433	DLB	Intepiridine	5HT-6 antagonist	Phase 2, DB-PC	UPDRS Part 3	Negative
NCT01023672	DLB	Armodafinil	Unknown	Open-label, pilot	ESS, MWT	Positive
NCT01340001	DLB	DBS of nucleus basalis of Meynert	Neuromodulation	Open-label, pilot	Free recall on FCSRT	Completed no results yet
NCT02258152	PDD	SYN120	5HT-6/5HT-2A antagonist	Phase 2, DB-PC	CDR computerized cognition battery continuity of attention	Negative
NCT01701544	PDD	DBS of nucleus basalis of Meynert	Neuromodulation	Open-label, pilot	Abbreviated cognitive battery, safety	Safe but no cognitive improvement
NCT02640729	DLB or PDD with VH	Nelotanserin	5HT-2A antagonist	Phase 2, DB-PC, cross-over	Safety, UPDRS Part 3	Safe/well tolerated but no significant changes on endpoints
NCT03325556	DLB or PDD with psychosis	Pimavanserin	5HT-2A inverse agonist/antagonist	Phase 3, time to event	Time to relapse	Positive
NCT02708186	DLB or PDD with RBD	Nelotanserin	5HT-2A antagonist	Phase 2, DB-PC	RBD frequency	Negative

*Abbreviations*: *ADAS-Cog* Alzheimer’s Disease Assessment Scale-Cognitive Subscale, *ADCS-CGIC* AD Cooperative Study-Clinical Global Impression of Change, *CIBIC+* Clinician’s Interview-Based Impression of Change plus Caregiver Input, *DB-PC* double-blind, placebo controlled, *DLB* dementia with Lewy bodies, *ESS* Epworth Sleepiness Scale, *FCSRT* Free and Cued Selective Reminding Test, *LBD* Lewy body dementia, *MoCA* Montreal Cognitive Assessment, *MWT* Maintenance of Wakefulness Test, *PDD* Parkinson’s disease dementia, *RBD* REM sleep behavior disorder, *UPDRS* Unified Parkinson’s Disease Rating Scale, *VH* visual hallucinations

## Optimizing clinical trial design in LBD (Table 2, Fig. 1)

### Study population

Fundamental considerations for LBD trials include whether to combine DLB and PDD participants in the same trial or to conduct separate trials for DLB and for PDD. Despite shared clinical, neurobiological, and pathological features, DLB and PDD groups may demonstrate differing treatment responses. DLB patients receiving memantine showed greater improvement on AD Cooperative Study-Clinical Global Impression of Change (ADCS-CGIC) scores than those receiving placebo, but the PDD group failed to show benefit vs. placebo; additionally, the DLB, but not the PDD, group showed significant improvement on Neuropsychiatric Inventory (NPI) scores [28]. AD co-pathology represents an important source of clinically meaningful biological heterogeneity in LBD phenotype and may influence clinical response. The development of biomarkers and clinical-pathological correlations are needed to help separate clinical differences between PDD and DLB from underlying biological differences.

**Table 2:** Barriers and challenges to developing clinical trials in Lewy body dementia

Category	Barriers and challenges
**Trial focus**	- Need for both disease-modifying and symptomatic trials - Lack of studies focusing on the breadth of LBD symptoms, including non-cognitive outcomes
**Study population**	- Delays in LBD diagnosis - Heterogeneity of clinical symptomatology - Co-morbidities (e.g., cerebrovascular disease) - Concomitant medication use (e.g., cholinesterase inhibitors, antipsychotics, parkinsonian medications)
**Recruitment and retention**	- Cognitively impaired population - Lack of under-represented minorities in studies - Complex and long assessment batteries - Study procedures (e.g., lumbar puncture, imaging) - Caregivers with high degrees of burden and stress - Long travel distances to study centers - Retention of older adults with combined cognitive, behavioral, and motor symptoms
**Selection of outcome measures**	- Lack of LBD-specific outcome measures - Existing outcome measures designed more for use in AD trials - Optimal outcome for different symptoms is uncertain - Existing outcome measures often lack validated measurement properties for LBD (e.g., inter-rater reliability, sensitivity to change)
**Study execution**	- Medication effects on attention and alertness - Cognitive fluctuations, which may affect test performance - “On” and “off” timing in individuals with Parkinson’s disease
**Biomarkers**	- Lack of biomarkers of progression - Lack of established α-synuclein biomarkers (imaging, biofluid) - Biomarkers in DLB criteria focus on diagnosis rather than clinical trial use - Lack of biomarker standardization - Lack of availability or access to some biomarker studies (e.g., dopaminergic imaging, polysomnography, cardiac MIBG)

*Abbreviations*: *AD* Alzheimer’s disease, *LBD* Lewy body dementia

**Fig. 1:**
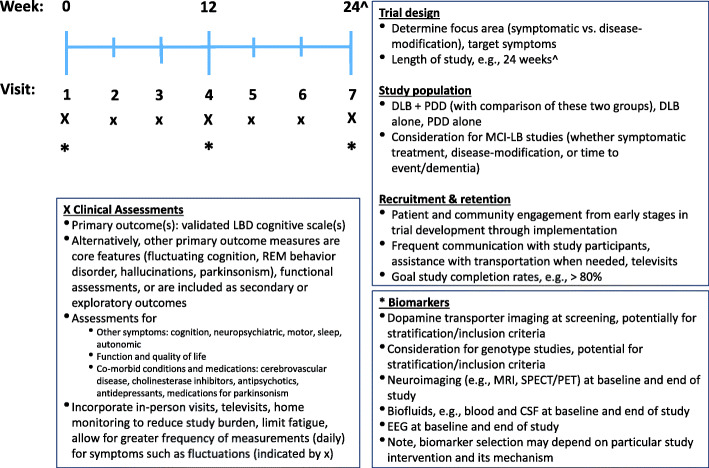
Considerations and an example for optimizing LBD clinical trial design. CSF, cerebrospinal fluid; DLB, dementia with Lewy bodies; LBD, Lewy body dementia; MCI, mild cognitive impairment; PDD, Parkinson’s disease dementia

Defining the LBD trial population requires decisions regarding which definitions and diagnostic criteria to use. To date, trials have primarily utilized DLB consensus criteria (3rd revision) [29] and the DSM-IV-TR for PDD. Very few trials have used the 2007 PDD criteria, and some trials utilize ranges or cutoff scores for the Mini-Mental State Examination (MMSE), Clinical Dementia Rating Scale, or Montreal Cognitive Assessment for enrollment [14]. Study criteria often do not specify the inclusion of probable vs. possible DLB. Defining PDD by DSM criteria may bias towards amnestic phenotypes, which may potentially reflect greater underlying AD co-pathology, and thus, influence treatment effects [30]. In LBD clinical trials, inclusion criteria have been based on clinical features rather than adjunctive biomarkers. In contrast, in some PD clinical trials, dopamine transporter scans are incorporated, and imaging and biofluid markers are commonly utilized in clinical trials for AD and the MCI stage of AD. With growing interest in disease-modifying strategies or symptomatic therapies for milder or earlier stages, how to best design trials for prodromal or MCI stages of PD (PD-MCI) or DLB (MCI-LB) merits consideration. While several trials have been conducted in PD-MCI, operationalization of MCI-LB research criteria is just beginning [17, 31–33].

Regarding the broad range of LBD symptomatology, the cognitive phenotype includes attentional, executive function, and visuospatial deficits, but memory and naming may also be affected in a subset of patients. Some LBD patients have greater neuropsychiatric, autonomic, or sleep phenotypes, and a subset of people with DLB do not have parkinsonism. Trials focusing on cognitive endpoints may not capture other LBD core or supportive features. Some trials specifically exclude major depression or capture additional LBD symptoms only in secondary or exploratory endpoints (e.g., NPI, Unified PD Rating Scale [UPDRS] Part 3). Detailed descriptions of core and supportive features in LBD trial participants are often lacking. One exception is the DLB donepezil trial by Mori et al. in which frequencies of core features were provided, along with details of suggestive and supportive features [34]. Co-morbidities such as cerebrovascular disease are variably described in LBD trials; such data were provided in the clinical trial for memantine but not rivastigmine [28, 35]. Concomitant medications frequently used in LBD (e.g., cholinesterase inhibitors, dopaminergic medications, antidepressants, and antipsychotics) also vary in how they are handled in study design. Some studies exclude LBD participants taking these medications, whereas others demonstrate variable rates of use, which potentially could influence outcomes and generalizability of findings. In memantine trials, cholinesterase inhibitors were excluded by Emre et al., but allowed by Aarsland et al. with their use in 47% of drug and 63% of placebo groups [28, 36]. Future trials may benefit from addressing a range of LBD symptoms, considering subgroups of LBD based on phenotypes, biomarker profiles, or genotype, as well as accounting for co-morbidities or co-pathologies that may influence cognitive status.

### Recruitment and retention

Recruitment and retention of study subjects are well-known challenges of clinical research, in general and in LBD. Subjects meeting criteria for many studies are often younger, male, healthier, wealthier, and more likely Caucasian, which may limit generalizability of findings. The study cohort in the Dubois et al. trial of donepezil in PDD, recruiting from 13 countries in Europe, plus Russia, South Africa, Australia, New Zealand, and Canada, was represented by 98% White, 1% Black, and 1% other [37]; similar demographics (98–100% White) are found in the LBD memantine and rivastigmine trials involving multiple European countries [28, 35]. Strategies for minority recruitment in LBD trials are needed. Subject retention and study completion can be difficult, with completion rates of about 70–80% in several LBD trials. These aspects can be particularly challenging when trials involve older participants with dementia, behavioral, and motor issues; assessment batteries are long and complex; procedural burden (blood draws, lumbar punctures, imaging) is high; and logistic issues (e.g., transportation, lodging, financial, fatigue) pose challenges. LBD caregivers, who also provide data for clinical trials, often face high degrees of burden and stress. Partnerships with patient advocacy groups and foundations for registries and awareness provide opportunities to help with study recruitment [38]. Considerations for transportation, televisits, or home monitoring may reduce barriers of study enrollment and retention [39, 40].

### Primary outcome measures

Determining primary outcome measures for LBD clinical trials include critical decisions such as whether to have a single primary or a co-primary outcome measure and whether to incorporate functional outcome measures or psychosocial aspects such quality of life or caregiver burden. These decisions may depend on the trial’s focus—symptomatic treatment vs. disease modification, certain clinical features, safety, tolerability, time to event, survival, phase of the trial (e.g., phase 1–4), and design (e.g., open label; double-blind, placebo-controlled trial; or other). Studies for approval of AD drugs typically have used a co-primary approach to assess global cognitive and functional measures. In 2018, the US FDA issued a new guidance that recognized the challenges in this approach, particularly for early stages of dementia, where drugs may target disease states prior to the onset of overt dementia [41]. This guidance proposed that for those with early stage AD, sensitive neuropsychological tests could be used as a primary endpoint to provide adequate support for drug approval, and some trials could utilize a time to event model for clinically meaningful events. Application of these guidances in LBD trial design awaits exploration and may carry relevance for targeting early stages of LBD including idiopathic RBD, MCI-LB, or PD-MCI [16, 17].

Primary endpoints of LBD trials, to date, have largely been AD-centric. Large, double-blind randomized trials for donepezil and rivastigmine in PDD used the AD Assessment Scale-Cognitive Subscale (ADAS-Cog) as the primary endpoint with the co-primary endpoint of the Clinician’s Interview-Based Impression of Change plus Caregiver Input (CIBIC+) or ADCS-CGIC, respectively [35, 37]. Two LBD memantine trials utilized the ADCS-CGIC [28, 36]. The MMSE and CIBIC+ were the primary endpoints in the randomized controlled trial of donepezil in DLB [34], and in another study, with the co-primary of the NPI-2 subscale for hallucinations and fluctuations, which has not been validated as an outcome measure in this form [42]. Several recent or ongoing trials in LBD utilize the Cognitive Drug Research (CDR) Computerized Assessment System, mainly attention scores (NCT03358253 with SYN120, NCT03305809 with LY3154207) [43].

Few LBD clinical trials have examined visual hallucinations, sleep (RBD, hypersomnolence), and motor signs of parkinsonism. Trials for autonomic symptoms, mood disorders, or fluctuations in LBDs are lacking. For those LBD studies of psychosis, primary endpoints vary from motor function (nelotanserin, NCT02640729), time to relapse of psychosis (pimavanserin, NCT03325556), and the NPI-psychosis subscale (MP-101, NCT03044249). A trial of nelotanserin for LBD-related RBD assessed RBD frequency (NCT02708186), and in an open-label, pilot study of armodafinil for hypersomnolence in DLB, primary efficacy measures were the Epworth Sleepiness Score and Maintenance of Wakefulness Test [44]. Studies of zonisamide in DLB for parkinsonism utilized the UPDRS Part 3 as the primary endpoint [45, 46].

Opportunities for LBD trial design include having endpoints and measures validated in LBD and that meet regulatory agency clinical trial qualifications. Such measures could be developed through data mining from longitudinal observational studies and clinical trials or from new or modified assessment instruments with appropriate psychometric properties for LBD. This will allow for modeling of effect size and appropriate duration of trials, both of which are critical for power estimates.

### Assessments

Testing for cognitive impairment for LBD trials requires properly validated measures, with strong test-retest reliability, sensitivity to the type of deficits and spectrum of cognitive impairment/dementia in LBD, and robust normative data. Decisions about cognitive test batteries need to consider global vs. specific domain assessments, length, burden, fatigue, fluctuations, and motor demands as well as floor and ceiling effects. Study design may factor in use of composite scores, measures of global cognition, and controlling for motor effects, even with computerized tests [47, 48].

While scales to elicit neuropsychiatric and autonomic features, collectively or individually, exist in the PD arena, validation in LBD is lacking. Trials must either focus only on selected features or balance the need to be comprehensive using a variety of different scales while minimizing potential study burden. Parkinsonism has typically been measured by the UPDRS Part 3 or MDS-UPDRS Part 3, but these clinical measurements do not fully capture the sensitivity and type of motor symptoms (e.g., gait, balance, falls) in LBDs or everyday motor function at home. Opportunities to assess motor features and response to intervention in broader, more continuous, and real-life environments may include use of quantitative measures and remote assessment with wearable sensor technology [49, 50].

Fluctuating cognition and alertness are core features in LBD that have the potential to greatly influence study outcomes, remain poorly understood, and are challenging to measure. To date, there are no established methods by which to account for DLB fluctuations in clinical trials, though several clinical scales, electrophysiological or imaging studies, or reaction time measurements may provide opportunities to assess this [51, 52]. Test performance at study visits may be affected by cognitive or physical fatigue, medications that alter attention and alertness, co-morbid psychiatric symptoms, orthostatic hypotension, and “on and off” timing in those with PD. Longitudinal data from observational studies and multiple data points obtained in interventional studies may help address these concerns and detect patterns of performance.

### Biomarkers and genetics

Biomarkers in LBD, whether neuroimaging (molecular, structural, functional), fluid-based (blood, cerebrospinal fluid [CSF], urine, or other), or electrophysiological, as well as genetic subtypes of LBDs have the potential to be used in research for patient selection, outcome measures, and target engagement [53]. Biomarkers in the DLB criteria aid in diagnosis and distinction from AD, though these biomarkers vary in their availability for clinical and research use [29]. For PDD diagnosis, the use of biomarkers remains limited to research. Several proteins have been investigated as biomarkers for LBD, including α-synuclein, amyloid-β, and tau, as measured by molecular imaging and biofluids and representing indirect measures of underlying neurodegeneration and pathology. While dopamine transporter scanning has been utilized in some PD trials for inclusion criteria, these scans have not been incorporated into LBD trials for patient selection [54]. Use of dopamine transporter imaging in early LBD (e.g., MCI-LB) also awaits further study. Stratifying LBD patients in clinical trials by dopaminergic imaging results may not be a simple decision as this may introduce bias towards more parkinsonian phenotypes of LBD. The lack of α-synuclein imaging remains a major gap in the field. Although amyloid-β PET imaging reveals that about 30–50% of people with LBD have positive scans, the frequency of positive tau imaging may be lower [55, 56]. This adds to the pathologic heterogeneity of LBD, since AD co-pathology is present in about 50% of autopsied LBD. Moreover, there is evidence of a dose-dependent association with survival [57]. Amyloid-β and tau biomarkers have potential diagnostic utility in LBD trials by allowing for subgroup stratification (e.g., LBD with or without co-existing AD) as well as in target engagement for disease-modifying trials. Biomarker profiling approaches in LBD would be greatly enhanced by having robust markers of α-synuclein pathology.

CSF measures of amyloid-β 1-42 (AB42), tau, and phosphorylated-tau have been associated with worse cognitive outcomes in some LBD studies [53]. CSF biomarkers, including α-synuclein and amyloid-β, could be used for study entry criteria and target engagement in LBD trials, though inclusion of lumbar punctures may affect subject enrollment and retention. One challenge is the high degree of variability in biomarker fluid assays including CSF; inter-laboratory variability for assays of α-synuclein is about 10% (range 5–20%) [58]. Thus, standardizing fluid analyses is essential. Longitudinal data for many of these biomarkers as well as comparisons between DLB and PDD, and among subgroups or at-risk cohorts (e.g., RBD, MCI), are crucial. Incorporation of amyloid-β biomarkers (imaging and/or CSF) into inclusion criteria is already underway in AD trials, as are trials of anti-amyloid therapeutics [59, 60]. In LBD, one study of memantine incorporated biomarkers, finding that some PDD receiving drug and having high homocysteine levels responded significantly better [61]. Opportunities include partnerships between AD and LBD biomarker efforts, data and sample sharing (e.g., federal- and foundation-funded programs), development and continued follow up of well-characterized clinical cohorts with biomarker samples (e.g., PD Biomarker Program, Parkinson’s Progression Markers Initiative, NIA AD Centers, DLB consortium), and autopsy studies.

EEG is recognized as a supportive biomarker in the diagnosis of DLB, with prominent posterior slow-wave activity with periodic fluctuations in the pre-alpha/theta range [13]. Quantitative or visually assessed EEG differentiates DLB from AD with high sensitivity and specificity and may correlate with cognitive changes, fluctuations, and hallucinations in DLB [62, 63]. EEG also may have a role not only in diagnosing MCI-LB, with slower frequencies in pre-alpha and theta ranges compared to healthy controls and intermittent delta activity patterns differentiating MCI-LB from MCI-AD, but also in predicting conversion from MCI-LB to DLB [64, 65]. While potentially less costly and less invasive than some biomarker studies, use of EEG as a diagnostic or prognostic biomarker for LBD will require increased standardization, understanding of inter-individual variability, integration with imaging and biofluid markers, and larger-scale prospective studies.

Genetic risk factors for both AD and PD may contribute to the development and symptoms of LBD. Some genetic mutations or polymorphisms (e.g., *APOE* locus, *MAPT*, and *GBA* genes) may influence the presence and severity of cognitive impairment in LBD [66–68]. *APOE e*4 carrier status has been linked to greater cognitive impairment and co-existing AD pathology in LBD [57, 67, 68]. To date, these genetic markers have not been utilized in LBD trials. In contrast, AD/MCI trials have incorporated genetic mutations (e.g., dominantly inherited AD) or polymorphisms (*APOE e*4 alleles) into trial design. *GBA* mutations, a susceptibility factor for PD, are found in those with DLB and LBD with AD pathology to a greater degree than in AD or control participants [67]. Trials targeting GBA carriers in PD are emerging [69]. In addition, GBA may play a role in target engagement for trials. Ambroxol is currently under study in a phase 2 trial of PDD (NCT02914366), examining its effect on the ADAS-Cog and CGIC, with additional measures of MRI, CSF, and Gcase activity in lymphocytes [70].

## Conclusions

In summary, there are challenges but also opportunities for clinical trials in LBD. Advances in our understanding of LBD symptomatology, diagnoses, prodromal stages, longitudinal changes, and pathophysiological mechanisms will enable us to discover new targets and strategies for therapeutic interventions. This approach will enable us to define more biologically homogenous groups of LBD patients for inclusion criteria that can improve efficiency of trials, especially those targeting specific underlying disease mechanisms for α-synuclein and/or AD neuropathology. Defining trial focus areas with input from the LBD patient community, along with implementing outcome measures validated in LBD; comprehensive, but not burdensome, test batteries; and biomarkers reflecting key mechanisms of disease are future goals of LBD trials. Mock clinical trials, as performed in frontotemporal lobar degeneration, may provide insight into disease progression and optimal outcome measures [71]. Observational clinical research in PD, PDD, and DLB with harmonized assessments, brain donation for research, and data and biosample sharing are critical. Partnership and consultation across patients, clinicians, researchers, industry, and government agencies on trial design and regulatory guidance regarding FDA will be vital for the future of LBD trials.

## Data Availability

NA

## Abbreviations

AD: Alzheimer’s disease
ADAS-Cog: AD Assessment Scale-Cognitive Subscale
ADCS-CGIC: AD Cooperative Study-Clinical Global Impression of Change
APOE: Apolipoprotein E
CIBIC+: Clinician’s Interview-Based Impression of Change plus Caregiver Input
DLB: Dementia with Lewy bodies
DSM: Diagnostic and Statistical Manual of Mental Disorders
FDA: Food and Drug Administration
GBA: Beta-glucosidase
IAC: Industry Advisory Council
LBCRS: Lewy Body Composite Risk Score
LBDA: Lewy Body Dementia Association
MAPT: Microtubule-associated protein tau
MCI: Mild cognitive impairment
MCI-LB: Mild cognitive impairment-Lewy body
MMSE: Mini-Mental State Examination
NACC: National Alzheimer’s Coordinating Center
NPI: Neuropsychiatric Inventory
PD: Parkinson’s disease
PDD: Parkinson’s disease dementia
PD-MCI: Parkinson’s disease-mild cognitive impairment
RBD: REM sleep behavior disorder
RCOE: Research Centers of Excellence
UPDRS: Unified PD Rating Scale
